# Association between high-mobility group box 1 levels and febrile seizures in children: a systematic review and meta-analysis

**DOI:** 10.1038/s41598-023-30713-w

**Published:** 2023-03-03

**Authors:** Shangbin Li, Qian Zhao, Jingfei Sun, Weichen Yan, Jie Wang, Xiong Gao, Xueying Li, Changjun Ren, Ling Hao

**Affiliations:** 1 Department of Pediatrics, The First Affiliated Hospital of Hebei Medical University, Shijiazhuang, Hebei China; 2 Department of Pediatrics, Zhengding people’s Hospital, Shijiazhuang, Hebei China

**Keywords:** Medical research, Neuroscience

## Abstract

The relationship between High-mobility group box 1 (HMGB1) and febrile seizures (FS) in children remains unclear. This study aimed to apply meta-analysis to reveal the correlation between HMGB1 levels and FS in children. Databases including PubMed, EMBASE, Web of science, Cochrane library, CNKI, SinoMed and WanFangData were searched for relevant studies. Pooled standard mean deviation and 95% confidence interval were calculated as effect size since the random-effects model was used when I^2^ > 50%. Meanwhile, between-study heterogeneity was determined by performing subgroup and sensitivity analyses. A total of 9 studies were finally included. Meta-analysis showed that the children with FS had significantly higher HMGB1 levels compared with healthy children and children with fever but no seizures (P<0.05). Additionally, subgroup analysis showed that the HMGB1 level in children with complex FS was higher than those with simple FS (P<0.05), and children with duration >15 min were higher than those with duration ≤15min (P<0.05). There were no statistical differences between children with or without a family history of FS (P>0.05). Finally, children with FS who converted to epilepsy exhibited higher HMGB1 levels than those who did not convert to epilepsy (P<0.05). The level of HMGB1 may be implicated in the prolongation, recurrence and development of FS in children. Thus, it was necessary to evaluate the precise concentrations of HMGB1 in FS patients and to further determine the various activities of HMGB1 during FS by well-designed, large-scale, and case-controlled trials.

## Introduction

Febrile Seizures (FS) are the most common form of childhood seizures, occurring in 2–5% of children between 6 months and 6 years old, and about 30–50% of cases may relapse after the first onset^1–3^. According to the 2011 American Academy of Pediatrics (AAP) standards, FS are defined as fever-induced (anal temperature ≥38.5 °C, axillary temperature ≥38 °C) seizures athat are not accompanied by a central nervous system infection, metabolic disorder and anamnesis^4^. Currently, no long-term and serious damage to children (including Children with FS infected with COVID-19) has been found, but long-term follow-up is required^5,6^. FS can be divided into simple FS and complex FS. Complex FS, which last for 15 min or more and can recur within 24 h, are related to focal nervous system manifestations^7^. Unfortunately, complex FS may develop into epilepsy^8^. In addition, there are no effective means to predict and intervene in febrile seizures into epilepsy. High mobility group protein 1 (HMGB1) is a nuclear DNA binding protein and is considered a new pro-inflammatory cytokine^9^. It has been extensively studied because of its function as an alarm protein that activates innate immunity. In recent years, more studies have found that the level of HMGB1 in the peripheral blood of children with FS was elevated. However, some studies also found that the level of HMGB1 in cerebrospinal fluid of children with FS is not statistically abnormal. Whether HMGB1 is involved in the development of childhood seizure is still unclear and controversial.

In this study, we systematically reviewed studies investigating the correlation between peripheral blood/CSF HMGB1 levels and FS in children.

## Methods

### Literature search

The PRISMA statement (an updated guideline for reporting systematic reviews) was followed in the systematic review and meta-analysis^10^. We searched for related articles and research published in the following electronic databases: PubMed, EMBASE, the Cochrane library, Scopus, Web of science, China National Knowledge Infrastructure (CNKI), Chinese Biology Medicine Disc (SinoMed) and WanFangdata (as of February 2, 2023). The terms applied for the database search included (“HMGB1” OR “High mobility group box 1”) AND (“febrile seizures” OR “FS” OR “febrile convulsion”). At the same time, relevant references are tracked to minimize missed detection rates. The search for correlational studies was not restricted to language, population, or publication year. The retrieval strategy of the PubMed database has been added to the Supplementary file.

### Selection of studies

The inclusion criteria include: (1) cross-sectional, case-control or cohort studies; (2) children diagnosed with FS (age <18 years) included in the study; (3) data on the expression level of HMGB1 in children with FS can be extracted;

The exclusion criteria were as follows: (1) Research population that was repeated in another study; (2) The study was published in the form of animal studies, reviews, abstracts, and letters. (3) Studies that did not measure HMGB1 levels, including pharmacology, genetics, brain imaging, and postmortem studies; (4) Unavailable Full text; (5) Unable to extract data.

### Data extraction and quality assessment

All data were independently extracted by two professionally trained investigators, with any disagreements resolved through discussion with another third investigator. All authors had no objection to the final results. The extracted data includes the first author’s name, publication year, study time, average age, the number of cases and controls, average and standard deviation (SD). The quality of the studies was estimated by the Newcastle–Ottawa Scale (NOS)^11^, which was evaluated as high (scored 7–9), medium (scored 4–6), or low (scored 0–3) quality.

### Statistical analysis

In Stata SE15.0 (StataCorp, College Station, TX), the standard mean deviation (SMD) and 95% confidence interval (CI) were selected to summarize and analyze the extracted data. Heterogeneity was determined by the I^2^ and Q statistics. When the p-value of the Cochrane Q test is <0.05 or I^2^ ≥50%, the random effects model was applied to calculate the SMD and 95% (CI). Otherwise, the fixed effects model was used. Based on the research design requirements, methods such as subgroup analysis and sensitivity analysis were used to test the stability of the combined effect. Publication bias was confirmed by Begg’s and Egger’s tests. The p-value<0.05 was regarded as a statistically significant standard.

## Results

### Literature search and screening

**Figure 1:**
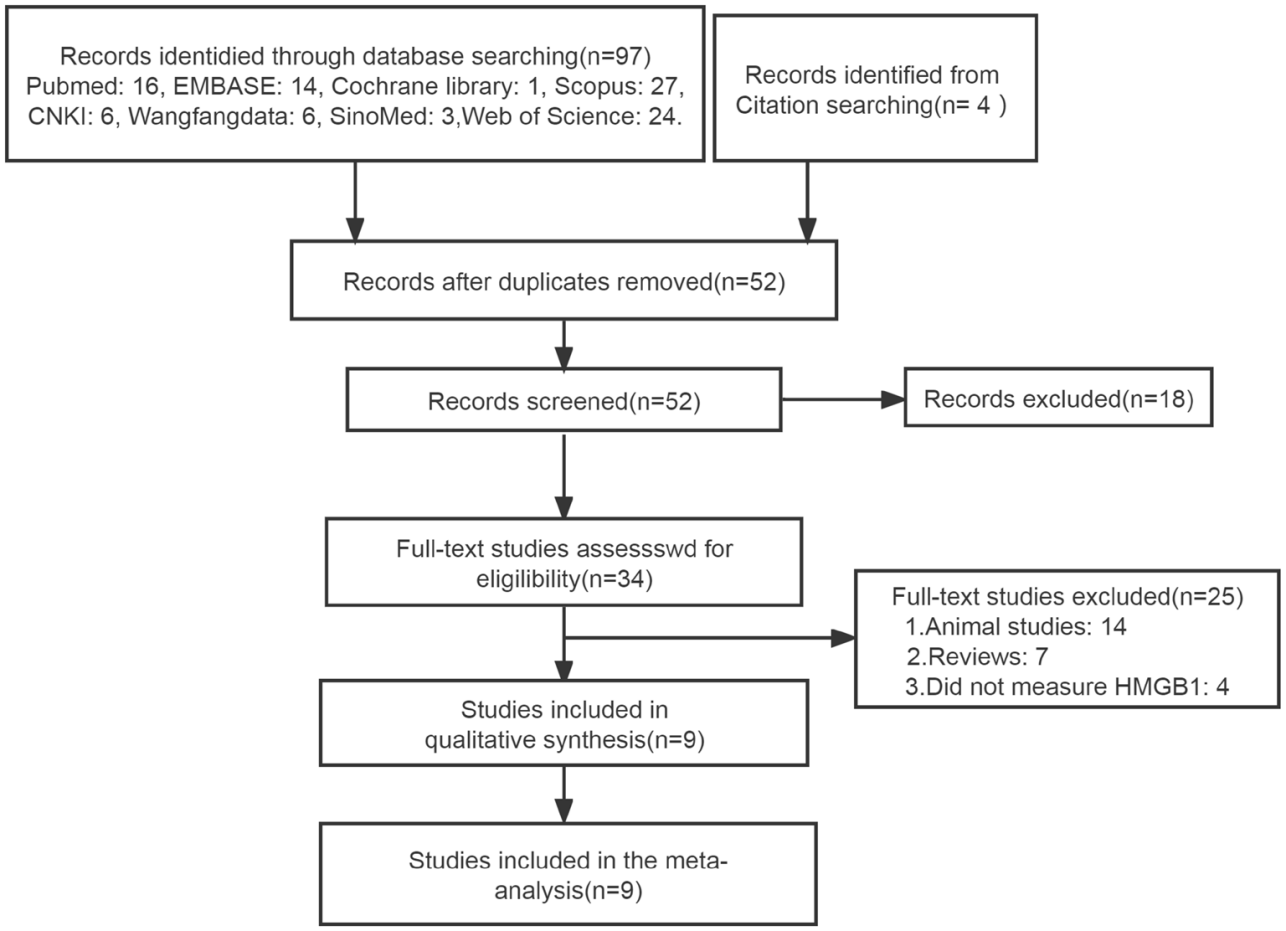
Flowchart of the literature search and study selection.

First, after removing 49 duplicate documents from 101 potential search studies, 18 unrelated studies were further excluded by the screen of title and abstract. Then, 14 animal studies, 7 reviews and 4 non-evaluation HMGB1 studies were removed from the remaining studies under careful review. Therefore, 9 studies^12–20^ that included 1494 children (including 867 children with FS, 316 children with only fever without FS, and 311 healthy control children) were included in the final meta-analysis. The flow chart of the search and study selection is shown in Fig. 1.

### Research characteristics

All included studies were published between 2011 and 2021. The eligible study sample size ranged from 24 to 559 participants, with the majority of the population coming from China, South Korea, Egypt, and Turkey. Among all included studies, a total of 7 studies investigated the HMBG1 relationship between children with FS and healthy control children^12–15,17–19^. Seven studies were based on peripheral blood results^13–19^, while 2 studies were based on cerebrospinal fluid^12,20^. Eight studies reported the expression levels of HMBG1 in children with FS and children with fever but no FS^12,14–20^, including 6 results based on serum and 2 results based on cerebrospinal fluid. Four studies reported the difference in serum HMBG1 between children with simple FS and children with complex FS^12–15^. Two studies reported the difference in serum HMBG1 between children in the FS converted to epilepsy group and children who did not convert to epilepsy^14,15^. A summary of all research characteristics is shown in Table 1. All included studies received a quality score ranging from 6 to 8, with an average score of 7.0.

**Table 1:** Characteristic of all included literature.

First author	Year	Country	FS/FN/control (number of samples)	Case/control age	Tissue	Detection methods	Time of collection	Type of study	Selection	Comparability	Exposure/result	Total score
Mehmet et al.^12^	2021	Turkey	40/13/45	All children: 6 months–6 years old	CSF	ELISA	Within the first 24 h after the seizure	Case-control	★★★★	★★	★★	8
Jinglin et al.^13^	2021	China	90/0/60	FS group: 34.04±9.54 months; healthy control: 35.45±10.32 months	BLOOD	ELISA	The FS group was collected on an empty stomach within 24 h after admission, and the healthy control group was collected on an empty stomach in the morning of the physical examination day	Case-control	★★★	★★	★	6
Dongxiu et al.^14^	2021	China	180/50/50	FS group: 2.14±0.62 years old; FN group: 2.08±0.59 years old; healthy control: 2.05±0.56 years old	BLOOD	ELISA	FS group and FN group were collected on an empty stomach within 24 h after admission (before treatment), and the healthy control group was collected on an empty stomach in the morning of the physical examination day	Case-control	★★★★	★★	★	7
Tingting et al.^15^	2020	China	359/100/100	All children: 3–60 months	BLOOD	ELISA	FS group and FN group were collected on an empty stomach within 24 h after admission (before treatment), and the healthy control group was collected on an empty stomach in the morning of the physical examination day	Case-control	★★★★	★★	★★	8
Choi et al.^16^	2019	Korea	38/20/0	All children: 3 months–5 years old	BLOOD	ELISA	Patients within 2 h of the time of seizure	Case-control	★★★★	★★	★	7
Marianne et al.^17^	2015	Egypt	50/51/25	FS group: 11.0 (6.0–50.0) months; FN group: 15.0 (6.0–51.0) months; healthy control: 9.0 (6.0–49.0) months	BLOOD	ELISA	Patients within 30 min of the time of seizure	Case-control	★★★★	★★	★★	8
Junhua et al.^18^	2013	China	53/33/24	FS group: 2.34±1.71 years old; FN group: 2.51±1.51 years old; healthy control: 2.22±1.37 years old;	BLOOD	ELISA	FS group and FN group were collected on an empty stomach within 24 h after admission (before treatment), and the healthy control group was collected on an empty stomach in the morning of the physical examination day	Case-control	★★★	★★	★	6
Choi et al.^19^	2011	Korea	41/41/7	All children: 6 months–6 years old	BLOOD	ELISA	Patients within 30 min of the time of seizure	Case-control	★★★★	★★	★	7
Takeshi et al.^20^	2011	Japan	18/6/0	All children: 11 months–10 years old	CSF	ELISA	Not mentioned	Case-control	★★★	★★	★	6

*FS* febrile seizures, *FN* fever but no convulsion, *CSF* cerebrospinal fluid, *ELISA* enzyme-linked immunosorbent assay.

### Meta analysis results

#### Meta-analysis of serum HMGB1 levels

**Figure 2:**
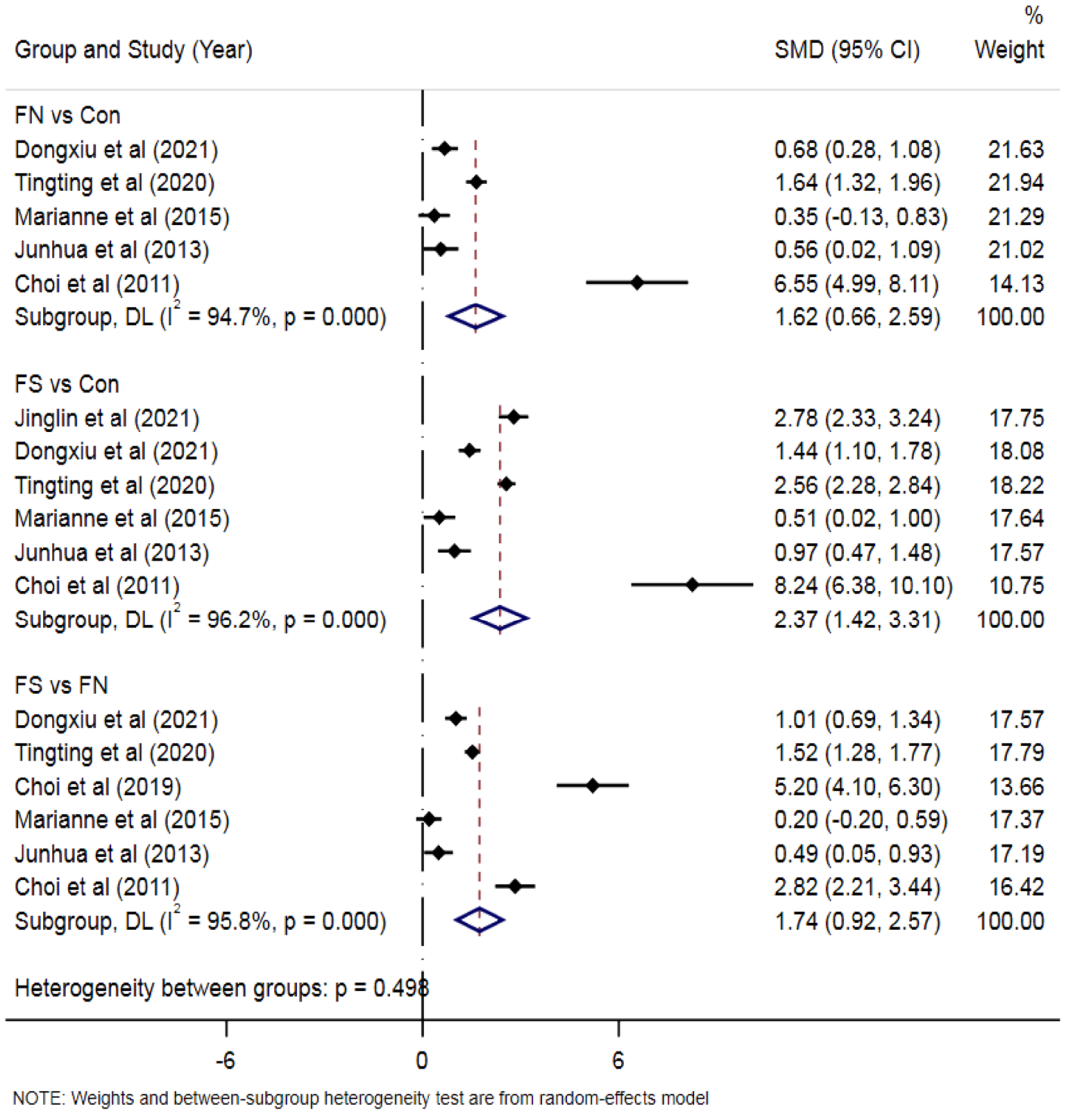
Forest plots of serum HMGB1 levels in FS group, FN group and healthy controls.

Serum HMGB1 levels were investigated in 5 case-control studies with 275 patients with FN and 206 healthy controls. Compared with the control group (Fig. 2), the FN patient group’s SMD was 1.62, the 95% CI was from 0.66 to 2.59, and P < 0.001. Furthermore, a combined analysis of the 6 included studies (including 773 patients with FS and 266 healthy controls) showed that the concentration levels of HMGB1 in the serum of children with FS were higher than that of healthy controls [SMD = 2.37, 95% CI  1.42–3.31; P< 0.001] (Fig. 2). Then, 6 studies (including 721 patients with FS and 295 healthy controls) were included in the serum HMGB1 meta-analysis. The FS group’s serum HMGB1 levels were significantly higher than that of children with fever and no seizures [SMD = 1.74, 95% CI  0.92–2.57; P < 0.001] (Fig. 2). However, due to the heterogeneity of the results, the random effects model was used for the meta-analysis of serum HMGB1 levels.

#### Meta-analysis of CSF HMGB1 levels

**Figure 3:**
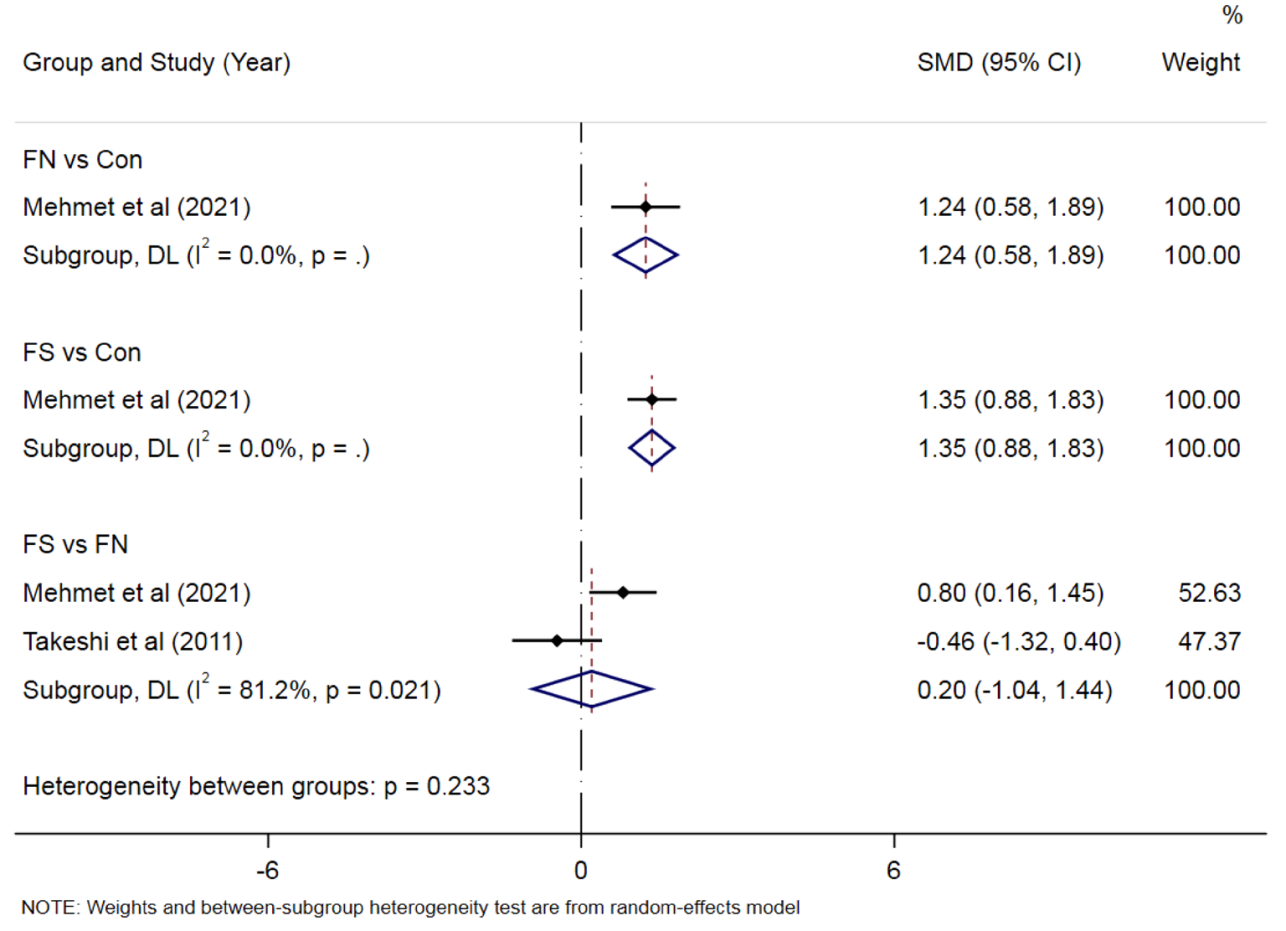
Forest plots of CSF HMGB1 levels in FS group, FN group and healthy controls.

There are few studies on HMGB1 levels in cerebrospinal fluid of children with FS. One study with 41 patients in the FN group and 13 patients in healthy controls showed that the FN group’s HMGB1 levels in CSF were higher than that of healthy controls [SMD =1.24, 95% CI  0.58–1.89; p < 0.001] (Fig. 3). In addition, the FS group’s levels were higher than those of the control group [SMD =1.35, 95% CI  0.88–1.83; P < 0.001] (Fig. 3). Fifty-six patients with FS and 21 patients in the FN group were extracted from 2 studies. However, the meta-analysis results showed that there was no significant difference in HMGB1 levels in CSF between the FS group and the FN group (P>0.05) (Fig. 3).

#### Sensitivity analysis and publication bias analysis

**Figure 4:**
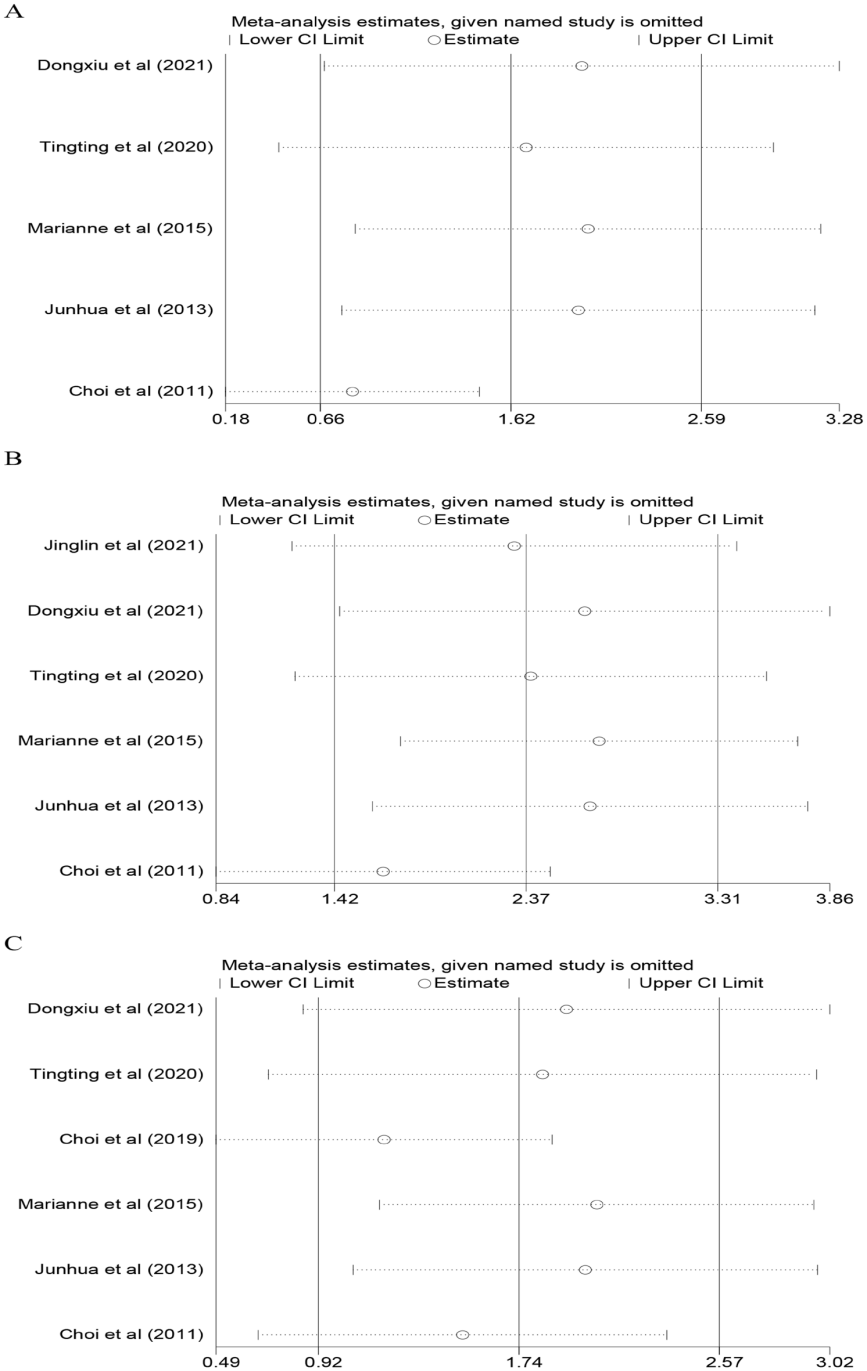
Sensitivity Analysis of all included studies ((**A**) FN group and healthy controls; (**B**) FS group and healthy controls; (**C**) FS group and FN group).

**Figure 5:**
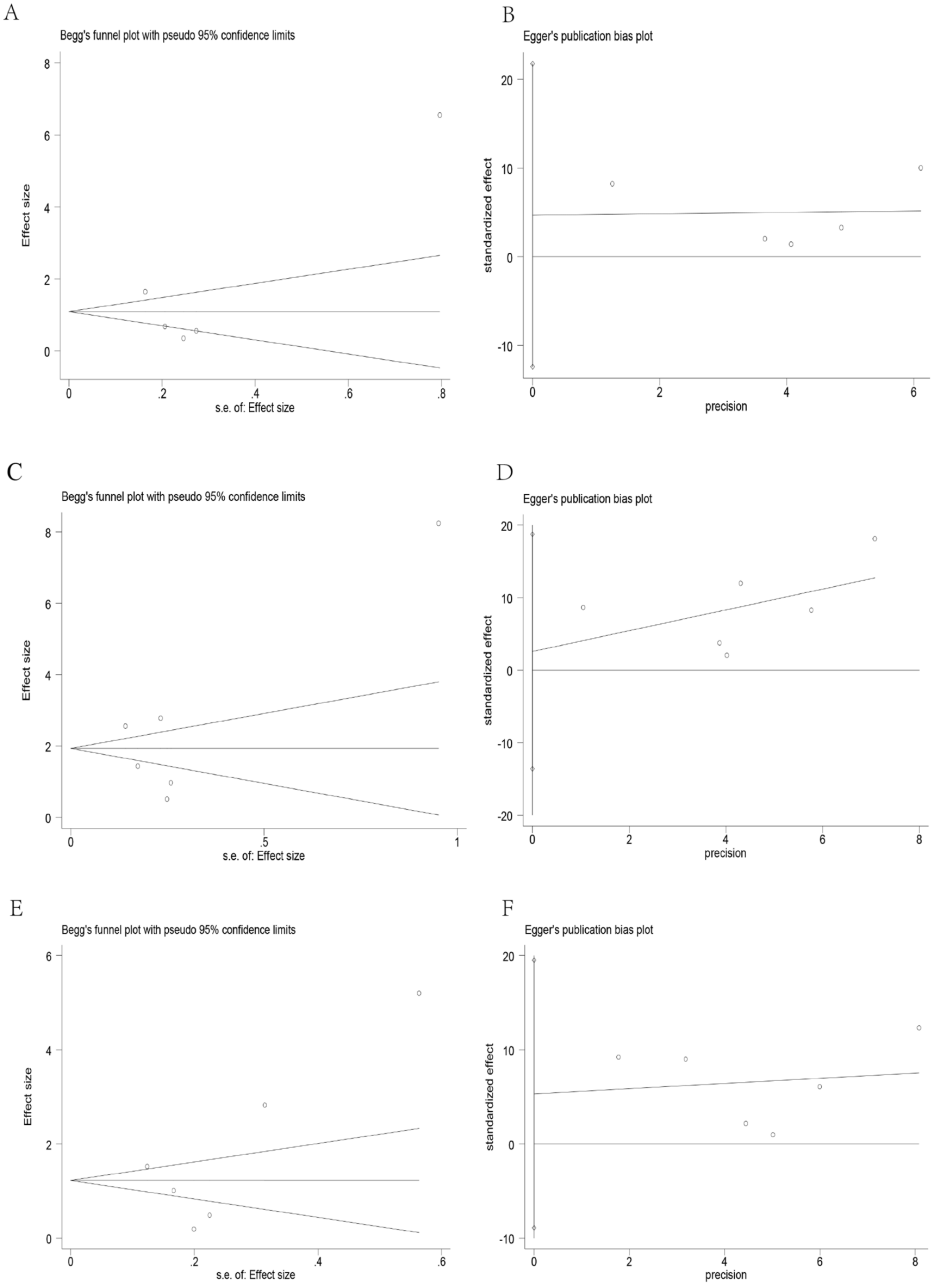
Publication bias tests of all included studies ((**A,B**) a funnel of Begg’s test and Egger’s test of FN group and healthy controls: (**C,D**) a funnel of Begg’s test and Egger’s test of FS group and healthy controls; (**E,F**) a funnel of Begg’s test and Egger’s test of FS group and FN group.

As shown in Fig. 4: sensitivity analysis was conducted by excluding individual studies. The results showed that the overall results of the three comparison groups were not significantly affected by particular studies. Publication bias was found using a funnel plot. However, the Begg-Mazumdar rank correlation test and Egger’s regression test did not show evidence of publication bias (Fig. 5). The studies included in the three comparison groups had no significant publication bias (FN group and healthy controls: Begg’s test (P = 0.806), Egger’s test (P = 0.447); FS group and healthy controls: Begg’s test (P = 1.000), Egger’s test (P = 0.681); FS group and FN group: Begg’s test (P = 0.452), Egger’s test (P = 0.357).

#### Subgroup analysis

According to the types and characteristics of seizures, further subgroup analysis (Table 2) showed that the levels of serum HMGB1 in complex FS were higher than that in simple FS (P < 0.05); The levels of serum HMGB1 in children with seizures duration > 15 min were higher than that of children with seizures ≤ 15 min (P < 0.05); There was no significant difference between family history and non-family history (P > 0.05). It is worth noting that the serum HMGB1 levels in children in the FS converted to epilepsy group were higher than that of children who did not convert to epilepsy (P < 0.05).

**Table 2:** Subgroup analysis of serum HMGB1 levels of different febrile seizure types, seizure types, seizure duration, and family history of seizures.

Types	No. studies	SMD (95% CI)	Heterogeneity	Effect model	P value
Types of FS					
Complex vs. simple	4	1.08 (0.67, 1.48)	76.5%	Random	<0.001
Duration of FS					
> 15 min vs. ≤ 15 min	2	0.97 (0.68, 1.26)	0%	Fixed	<0.001
Family history(FH)					
With FH vs. no FH	2	0.20 (− 0.20, 0.43)	47.2%	Fixed	>0.05
Conversion to epilepsy					
Yes vs. No	2	0.56 (0.34, 0.79)	45.9	Fixed	<0.001

## Discussion

This study is the first meta-analysis to evaluate the level of HMGB1 in children with FS. A total of 1494 children (including 867 children with FS, 316 children with FN, and 311 healthy control children) were included in 9 studies. The sample size was large and persuasive. This study first compared the differences between FS children, healthy children, and children with FN, then further compared different regions, sources of HMGB1 and types of seizure through subgroup analyses, as well as the difference between the duration of seizure and the family history of seizure. The sensitivity analysis shows that the results are consistent and reliable. In addition, Begg’s and Egger’s tests showed that all the included studies had no publication bias, and the conclusion were reliable.

Some studies believe that fever is a normal response to infection, and releasing high levels of cytokines during fever may change normal brain activity and cause seizures^21^. Meta-clinical evidence shows that the HMGB1 level of children with FN is higher than that of healthy control children. It can be considered that the level of HMGB1 in children with FN will increase due to the inflammation of the immune system caused by infection^22^. Furthermore, the level of HMGB1 in children with FS was higher than that in healthy control children, suggesting that fever or seizure or both of them caused the increase of HMGB1 level in children with FS. In a comprehensive analysis, the level of HMGB1 in children with FS was significantly higher than that in children with FN, which could be considered as an important cytokine mediator in the pathogenesis of FS in children, and it had the ability to distinguish whether there is seizure or not. However, the exact mechanism of the relationship between the HMGB1 level and the development of FS still needs to be fully understood.

HMGB1 is a late inflammatory protein released passively by necrotic tissue or actively secreted by stress cells^23^. In non-tumor diseases, HMGB1 triggers inflammation as a damage-related molecular model, and its pro-inflammatory effect is closely related to its release to the extracellular environment^24^. Extracellular HMGB1 can regulate cell migration and stimulate a variety of inflammatory and immune cells to produce cytokines such as TNF-α and IL-1β by binding to receptors for the receptor of advanced glycation endproducts (RAGE) and Toll-like receptors(TLR)^25,26^. Previous studies have confirmed that elevated cytokine levels during infection play a major role in fever in FS^27^. After hyperthermia-induced seizures in mice, the expression of HMGB1, IL-1β, TNF-α and other pro-inflammatory cytokines in the hippocampus and cortex increased significantly^28^. The activation of IL-1 receptor/toll-like receptor (IL-1R/TLR) signal reduces the seizure threshold and significantly promotes seizures^29^. A study confirmed that abnormally increased IL-1β The level will gradually increase excitatory (glutamate) neurotransmission and reduce inhibitory (GABAergic) neurotransmission, causing the increase of calcium ions in neurons, leading to seizures^30,31^. We speculate that HMGB1 may play a regulatory role that it indirectly participated in the mechanism of glutamat-ergic and GABA-ergic neurotransmission disorders and fever-mediated febrile seizure during inflammation by increasing the expression of IL-1β and other proinflammatory cytokines. Unfortunately, Brennan et al. administered HMGB1 blockers to febrile rat pups will not reduce the expression of downstream inflammatory cascade reaction and lead to unacceptable side effects^32^. Therefore, whether HMGB1 can be used as a therapeutic target to prevent the occurrence or progression of FS is still unknown.

In addition, this study also showed that subgroup analysis showed that the level of HMGB1 in children with FS in East Asia and the Middle East was higher than that in healthy control group and children with FN in the same region, indicating that the high level of HMGB1 in different populations might be the main risk factor for FS in children. The level of HMGB1 in children with complex FS is higher than that in children with simple FS, and that in children with FS with convulsion duration > 15 min is higher than that in children with seizure duration ≤ 15 min, indicating that high level of serum HMGB1 is positively correlated with the duration of febrile seizure in children, and children with high level of HMGB1 are more prone to complex FS. Some studies have shown that children have a relatively high risk of complicated FS followed by epilepsy. Clinical studies also found that FS children with high levels of HMGB1 may develop epilepsy^33^. HMGB1 has been proved to play a role in epileptogenic response as an inflammatory cytokine^34^. HMGB1 can enhance seizures induced by heat treatment in developing rats and secondary epilepsy associated with seizures induced by long-term heat treatment^35^. However, the exact mechanism of FS developing into epilepsy is still unclear. At present, there is no evidence that simple FS can cause structural damage to the brain^36^. A series of prospective studies have shown that children with long-term FS are at risk of hippocampal damage and hippocampal sclerosis^37,38^. Repeated FS have been proved to change the time coordination function of the hippocampus, but do not affect the spatial memory ability^39^.

Interestingly, the study found that the levels of HMGB1 in the cerebrospinal fluid of children with FS is not statistically significant compared with children with FN. Theoretically, if the levels of HMGB1 in the central nervous system increases, HMGB1 in the cerebrospinal fluid should be the most ideal detection index. It was not excluded that it has a certain relationship with different stages, course of disease and the way of samples are collected and handled. Since there are few studies on cerebrospinal fluid HMGB1 in children with FS, it is necessary to carry out more clinical studies to explore and confirm.

There still exist some limitations in this study. First of all, the heterogeneity test results show that heterogeneity exists among the included studies, which may affect the effectiveness of this study. Especially, this study conducts subgroup analyses to investigate the potential influencing factors of the results. Further subgroup analysis based on the disease region shows that the heterogeneity of the Middle East region group decreases, while the heterogeneity of the East Asia region group remains relatively stable, which means that regional differences may be a potential source of heterogeneity. However, other potential factors such as age, sex ratio, environmental and clinical characteristics, and different manufacturers of ELISA kits may also affect the level of HMGB1 in peripheral blood/cerebrospinal fluid. Second, there is no pre-registration agreement in this study, which may bring some bias, but our study is carried out strictly according to the plan; Third, because there is no corresponding original data in some literatures, we extract some data from statistical charts through software and these data based on approximation may be deviated from the actual situation. Notably, HMGB1 is an intranuclear protein. Depending on the detection technology and time, different disease states and clinical stages may affect the expression and secretion of HMGB1. Although the above factors impact the level of HMGB1 in peripheral blood/cerebrospinal fluid, more and more studies have reported the potential mechanism of the relationship between the level of HMGB1 in peripheral blood/cerebrospinal fluid and febrile convulsion (Supplementary Information).

## Conclusion

Based on integrated clinical data, this meta-analysis systematically revealed that the FS patients had significantly higher serum HMGB1 levels than the FN groups and control groups. The difference of HMGB1 in cerebrospinal fluid in patients with FS needs to be further confirmed. In addition, well-designed, large-scale, multi-factor, multi-region and case-controlled trials are needed to evaluate the precise concentrations of HMGB1 in FS patients and to determine the various activities of HMGB1 during FS.

## Supplementary Information

Supplementary Information.

## Data Availability

Data can be obtained from the corresponding author Changjun Ren upon reasonable request.

## Abbreviations

FS: Febrile seizures
FN: Fever but no convulsion (children with febrile illness, but without convulsion)
CSF: Cerebrospinal fluid
ELISA: Enzyme-linked immunosorbent assay
CI: Confidence interval
