# Publisher Correction: Association between high-mobility group box 1 levels and febrile seizures in children: a systematic review and meta-analysis

**DOI:** 10.1038/s41598-023-34109-8

**Published:** 2023-05-01

**Authors:** Shangbin Li, Qian Zhao, Jingfei Sun, Weichen Yan, Jie Wang, Xiong Gao, Xueying Li, Changjun Ren, Ling Hao

**Affiliations:** 1 Department of Pediatrics, The First Affiliated Hospital of Hebei Medical University, Shijiazhuang, Hebei China; 2 Department of Pediatrics, Zhengding people’s Hospital, Shijiazhuang, Hebei China

Correction to: *Scientific Reports* 10.1038/s41598-023-30713-w, published online 03 March 2023

The original version of this Article contained errors in the Results section, under the subheading ‘Research characteristics’,

“Five studies were based on peripheral blood results^13,14,15,16,17,18,19^, while 2 studies were based on cerebrospinal fluid^12,21^. Eight studies reported the expression levels of HMBG1 in children with FS and children with fever but no FS^12,14,15,16,17,18,19,20,21^, including 6 results based on serum and 2 results based on cerebrospinal fluid.”

now reads:

“Seven studies were based on peripheral blood results^13,14,15,16,17,18,19^, while 2 studies were based on cerebrospinal fluid^12,20^. Eight studies reported the expression levels of HMBG1 in children with FS and children with fever but no FS^12,14,15,16,17,18,19,20^, including 6 results based on serum and 2 results based on cerebrospinal fluid.”

The original Article has been corrected.

