# Supplementary material for: Association between high-mobility group box 1 levels and febrile seizures in children: a systematic review and meta-analysis
Source: Sci Rep. 2023 Mar 3;13:3619. doi: 10.1038/s41598-023-30713-w (PMC9983536; doi:10.1038/s41598-023-30713-w)
Supplement: Supplementary file 1 — Supplementary Information. [file 41598_2023_30713_MOESM1_ESM.docx]

#1 (((((((((((((((((((((((((((((((((seizures, febrile[MeSH Terms]) OR (Febrile Seizure[Title/Abstract])) OR (Seizure, Febrile[Title/Abstract])) OR (Febrile Seizures[Title/Abstract])) OR (Pyrexial Seizure[Title/Abstract])) OR (Pyrexial Seizures[Title/Abstract])) OR (Seizure, Pyrexial[Title/Abstract])) OR (Seizures, Pyrexial[Title/Abstract])) OR (Pyrexial Convulsion[Title/Abstract])) OR (Convulsion, Pyrexial[Title/Abstract])) OR (Convulsions, Pyrexial[Title/Abstract])) OR (Pyrexial Convulsions[Title/Abstract])) OR (Febrile Fit[Title/Abstract])) OR (Febrile Fits[Title/Abstract])) OR (Fit, Febrile[Title/Abstract])) OR (Fits, Febrile[Title/Abstract])) OR (Fever Convulsion[Title/Abstract])) OR (Convulsion, Fever[Title/Abstract])) OR (Convulsions, Fever[Title/Abstract])) OR (Fever Convulsions[Title/Abstract])) OR (Fever Seizure[Title/Abstract])) OR (Fever Seizures[Title/Abstract])) OR (Seizure, Fever[Title/Abstract])) OR (Seizures, Fever[Title/Abstract])) OR (Convulsions, Febrile[Title/Abstract])) OR (Convulsion, Febrile[Title/Abstract])) OR (Febrile Convulsion[Title/Abstract])) OR (Febrile Convulsions[Title/Abstract])) OR (Febrile Convulsion Seizure[Title/Abstract])) OR (Febrile Convulsion Seizures[Title/Abstract])) OR (Seizure, Febrile Convulsion[Title/Abstract])) OR (Seizures, Febrile Convulsion[Title/Abstract])) OR (Seizure, Febrile, Simple[Title/Abstract])) OR (Seizure, Febrile, Complex[Title/Abstract])

#2 (((((((((((HMGB1 Protein[MeSH Terms])) OR (HMG1[Title/Abstract])) OR (FM1 Gene Product[Title/Abstract])) OR (HMG-1 Protein[Title/Abstract])) OR (Box Protein 1, High Mobility Group[Title/Abstract])) OR (Amphoterin[Title/Abstract])) OR (HMGB1[Title/Abstract])) OR (HMG 1 Protein[Title/Abstract])) OR (Heparin-Binding Protein p30[Title/Abstract])) OR (Heparin Binding Protein p30[Title/Abstract])) OR (p30, Heparin-Binding Protein[Title/Abstract])

#1 AND #2
